# Edge‐enhancement densenet for X‐ray fluoroscopy image denoising in cardiac electrophysiology procedures

**DOI:** 10.1002/mp.15426

**Published:** 2022-01-18

**Authors:** Yimin Luo, Yingliang Ma, Hugh O’ Brien, Kui Jiang, Vikram Kohli, Sesilia Maidelin, Mahrukh Saeed, Emily Deng, Kuberan Pushparajah, Kawal S. Rhode

**Affiliations:** ^1^ School of Biomedical Engineering and Imaging Sciences King's College London London UK; ^2^ School of Computing Electronics and Mathematics Coventry University Coventry UK; ^3^ School of Computer Science Wuhan University Wuhan China

**Keywords:** cardiac electrophysiology procedures, convolutional neural network, denoising, edge enhancement, X‐ray fluoroscopy

## Abstract

**Purpose:**

Reducing X‐ray dose increases safety in cardiac electrophysiology procedures but also increases image noise and artifacts which may affect the discernibility of devices and anatomical cues. Previous denoising methods based on convolutional neural networks (CNNs) have shown improvements in the quality of low‐dose X‐ray fluoroscopy images but may compromise clinically important details required by cardiologists.

**Methods:**

In order to obtain denoised X‐ray fluoroscopy images whilst preserving details, we propose a novel deep‐learning‐based denoising framework, namely edge‐enhancement densenet (EEDN), in which an attention‐awareness edge‐enhancement module is designed to increase edge sharpness. In this framework, a CNN‐based denoiser is first used to generate an initial denoising result. Contours representing edge information are then extracted using an attention block and a group of interacted ultra‐dense blocks for edge feature representation. Finally, the initial denoising result and enhanced edges are combined to generate the final X‐ray image. The proposed denoising framework was tested on a total of 3262 clinical images taken from 100 low‐dose X‐ray sequences acquired from 20 patients. The performance was assessed by pairwise voting from five cardiologists as well as quantitative indicators. Furthermore, we evaluated our technique's effect on catheter detection using 416 images containing coronary sinus catheters in order to examine its influence as a pre‐processing tool.

**Results:**

The average signal‐to‐noise ratio of X‐ray images denoised with EEDN was 24.5, which was 2.2 times higher than that of the original images. The accuracy of catheter detection from EEDN denoised sequences showed no significant difference compared with their original counterparts. Moreover, EEDN received the highest average votes in our clinician assessment when compared to our existing technique and the original images.

**Conclusion:**

The proposed deep learning‐based framework shows promising capability for denoising interventional X‐ray fluoroscopy images. The results from the catheter detection show that the network does not affect the results of such an algorithm when used as a pre‐processing step. The extensive qualitative and quantitative evaluations suggest that the network may be of benefit to reduce radiation dose when applied in real time in the catheter laboratory.

## 1 INTRODUCTION

Minimally invasive cardiovascular catheterization procedures, in which catheters are inserted through small incisions, have an increasing role in the management of cardiovascular diseases (e.g., coronary, congenital, adult structural, and arrhythmias) due to their high success rates and low patient morbidity.^1^ X‐ray fluoroscopy is an indispensable tool in such interventional procedures, as it offers continuous screening and desirable visualization of catheters. However, X‐ray imaging inevitably involves ionizing radiation and exposure to this radiation poses a non‐negligible threat to both patients and healthcare staff.^2^ To increase safety, the X‐ray radiation hazards can be reduced by decreasing the X‐ray output. Low‐dose X‐ray fluoroscopy is the most common approach to monitor the progress of interventions. Fluoroscopy images obtained using lower X‐ray doses have decreased risks but increased noise and artifacts. Excessive noise and artifacts can compromise vital information in the images, which can impair clinical decision‐making.

To ensure acceptable image quality while keeping the X‐ray dose as low as possible, it is possible to use denoising techniques. An effective denoising algorithm for X‐ray fluoroscopy imaging should increase signal‐to‐noise ratio (SNR) whilst preserving structures of interest, such as anatomical borders and devices. It should also be fast enough to allow real‐time implementation. There have been several attempts for X‐ray fluoroscopy denoising, ranging from conventional filter‐based methods to more recent learning‐based methods. Conventional filter‐based denoising methods can be applied in both the spatial and temporal domains. For example, the authors in references ^3^ and ^4^ proposed the use of Karhunen–Loève and wavelet transforms, respectively, in the temporal domain for denoising X‐ray fluoroscopy images. In reference 5, an adaptive spatio‐temporal filter based on the local conditional average of similar pixels was designed and showed acceptable performance on both synthetic and real data. Furthermore, to improve the segmentation of the objects in multi‐view fluoroscopy frames, the authors in reference 6 proposed a denoising algorithm based on directional binary masks to enhance the separability of curvilinear structures. A curvelet‐based spatial filter associated with a first‐order temporal filter was developed in reference 7, however, the obtained X‐ray fluoroscopy sequences sustained a motion blur during real‐time denoising which would limit the real‐word applicability. In order to make better use of both spatial and temporal information, they further proposed a spatio‐temporal filter which operates in a multi‐scale dimension.^8^ Despite the small computational cost of these methods, they are prone to produce over‐blurry sequences due to limited samples for reference. To make better use of prior knowledge, learning‐based methods have been further proposed and developed. After the rise of deep learning theory in recent years, convolutional neural networks (CNNs) have gradually begun to dominate the image denoising field due to their impressive potential for learning representation from visual data.^9^, ^10^, ^11^, ^12^ For example, the Denoising Convolution Neural Network (DnCNN) proposed in reference 10 is a benchmark for denoising of photographic and videographic images. Recently, CNN‐based frameworks^13^, ^14^ have been proposed for X‐ray fluoroscopy denoising. For example, the authors in reference 13 proposed a simple CNN‐based framework to simulate the nonlinear mapping between low‐dose and higher‐SNR X‐ray fluoroscopy image patches. The authors in reference 14 compared different existing CNN‐based frameworks on clinical and phantom data. Although, these methods lose some detail, quantitative and qualitative analyses have demonstrated that deep learning‐based approaches outperform well‐established conventional X‐ray image denoising methods.

Inspired by the progress of the use of dense connections^15^ on information reuse in CNN frameworks, we proposed a multiple‐path residual block, namely ultra‐dense block (UDB), for feature representation and designed a denoising framework stacked with multiple UDBs, namely the ultra‐dense denoising network (UDDN) in our previous work.^16^ We demonstrated that this framework can visually remove noise on low‐dose X‐ray fluoroscopy images and obtain a higher SNR when compared to several state‐of‐the‐art denoising methods, for example, DnCNN. However, in our assessments, the high‐frequency details (e.g., image edges) in the denoised images were reported as too smooth by cardiologists. To alleviate this problem, more attention should be given to edge information during CNN‐based denoising. Attention mechanisms which aim to extract specific features for various image processing applications have become a topic of interest in the current deep learning research field and have been widely used in many medical image segmentation tasks.^17^, ^18^, ^19^ For image denoising problems, the authors in reference 20 integrated an attention mechanism into a CNN to remove blind noise. So far there have been few attempts to apply attention mechanisms to medical image denoising tasks such as low‐dose X‐rays, and edge information has not been paid special attention during model optimization. Motivated by this, in this paper, we design an attention‐awareness edge‐enhancement module to increase edge sharpness and propose a novel CNN‐based denoising framework, namely edge‐enhancement densenet (EEDN). This framework consists of two main subnetworks: an initial denoiser and an edge‐enhancement network which is constructed to enhance the image contours by optimizing the edge map via an attention mechanism. Accordingly, the initial denoised result and the enhanced edge map can be combined to generate a composite output image.

Unlike segmentation algorithms, where effectiveness can be evaluated through accuracy, the evaluation of denoising performance on real‐world data is a challenging problem with high subjectivity. Besides visual perception, peak signal‐to‐noise ratio (PSNR) and structural similarity index measure (SSIM) are usually used as quantitative evaluation metrics to assess the model performance on image denoising tasks.^9^, ^10^, ^11^, ^12^, ^13^, ^14^, ^20^ However, both of these need *reference*/*ground truth*/*clean* images to validate the effectiveness of the obtained model. In real interventional procedures, we have only X‐ray fluoroscopy images with no corresponding ground truth. We chose local SNR to evaluate the denoising performance of our network on a total of 3262 frames from 100 low‐dose X‐ray fluoroscopy sequences acquired during 20 cardiac pacing studies. Next, to evaluate the edge restoration ability of EEDN, we compared the results of EEDN to the previous UDDN using frequency spectrum analysis. We performed a clinical evaluation using assessment by cardiologists via pairwise fluoroscopy sequence voting and feedback. In addition, we have applied catheter detection to the output of our framework using 416 images frames from 8 X‐ray fluoroscopy sequences acquired during 5 left atrial radio‐frequency ablations. These images contained coronary sinus (CS) catheters which were automatically detected to assess the effect of our network as a pre‐processing step for algorithms that rely on high‐frequency content.

## 2 METHODS

The goal of this framework is to learn a nonlinear mapping function *f* between X‐ray image patches from low‐ to pseudo‐high‐dose X‐ray images. Accordingly, to establish this nonlinear mapping, sufficient low‐ and high‐dose X‐ray image pairs are required as input and output, respectively, and an effective framework can be designed. In this section, we present the methodology for our X‐ray fluoroscopy sequence denoising framework, including the preparation of training data, the overall framework of EEDN and details on its attention‐awareness edge‐enhancement module. We provide four complementary evaluation methods to assess this framework.

### 2.1 Noise simulation

**FIGURE 1:**
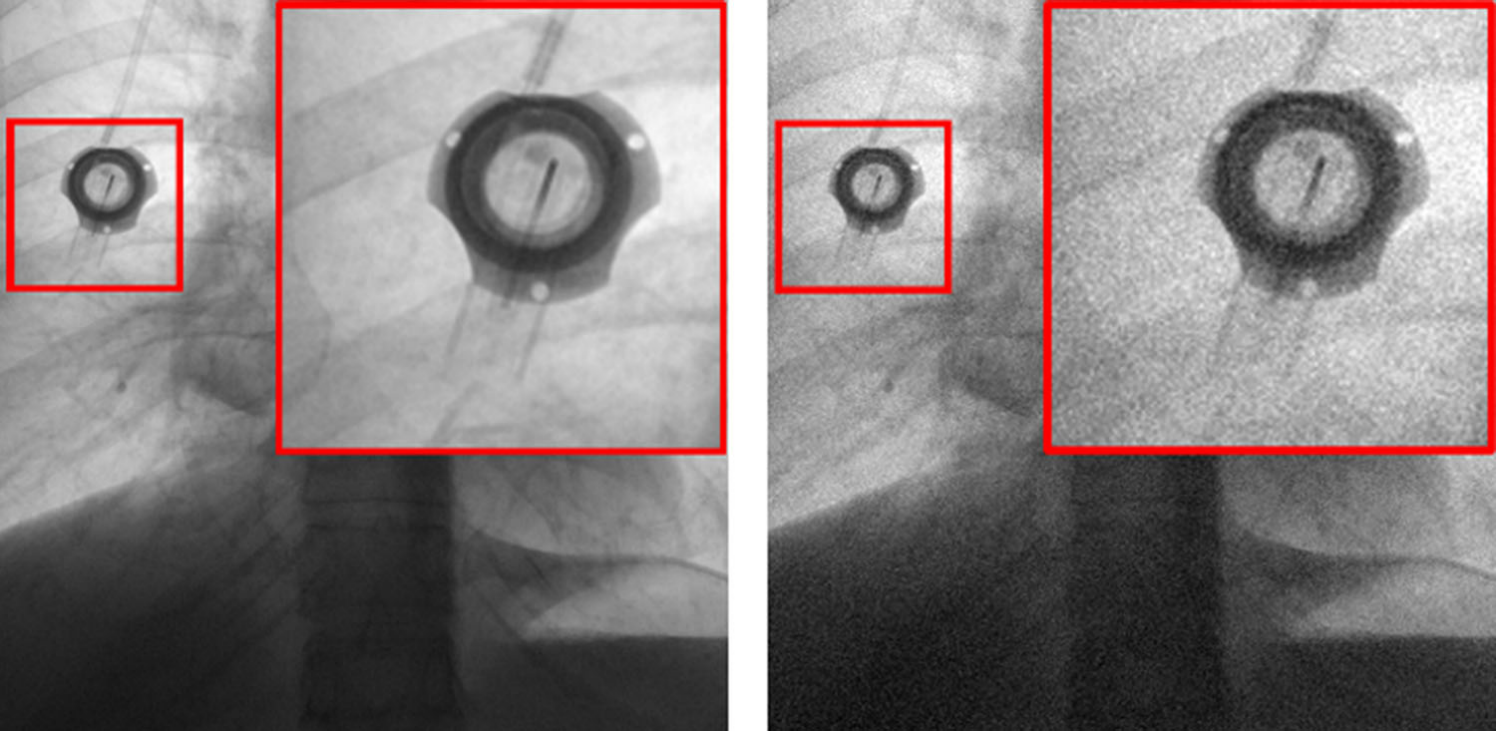
An example of adding simulated noise to a chest X‐ray image. Left: Image acquired at a relatively high dose. Right: Image with 60% simulated added noise

There are several sources of noise in an X‐ray image obtained using a digital detector. These include quantum noise (both from primary and scattered photons), electronic noise, and digitization noise. At the lowest image dose settings that are typically seen in cardiovascular catheterization procedures, the noise is quantum limited and follows a Poisson distribution. We first add synthetic Poisson noise to relatively high‐dose X‐ray images (ground truth) *I*’ to generate their low‐dose X‐ray counterparts as follow:

(1) $$I_{\mathrm{input}}\left(u,v\right)=I^{\prime }\left(u,v\right)+\mathrm{Poisson}\left(\lambda \right)$$

where *I*_input_ (*u*, *v*) is a pixel in the noisy image, *I*’ (*u*, *v*) is the corresponding pixel in the ground truth image; Poisson(λ) is a random number generated from a Poisson distribution with mean *λ *= *μα*/100; *μ* is the percentage noise level and *α* is the mean intensity value of all pixels in *I*’. Figure 1 shows a chest X‐ray image taken from a publicly available chest X‐ray dataset, ChestX‐Ray8^21^ (CXR), with additive synthetic noise going from the original image (*α* = 0%) to *α* = 60%.

On a cardiac catheterization X‐ray system, there are two‐foot pedals used to control which exposure is used. In this paper, we refer to the low‐dose exposure as fluoroscopy and the other, higher‐dose exposure, as acquisition. The actual dose settings are pre‐programmed by the service engineer and are particular for different procedures and operator preferences. According to an empirical observation at our institution, the difference seen between the acquisition and the fluoroscopy mode for cardiac electrophysiology procedures would correspond to appropriately 60% additive noise. Previous denoising methods for natural images presented in references 9, 10, 11 and ^20^ usually use random or fixed noise levels for training, which does not match the noise level of the X‐ray fluoroscopy sequences used in clinical practice. Therefore, to promote the clinical applicability of our CNN‐based framework to a variety of X‐ray systems and system settings, Gaussian‐distributed noise variation was proposed in reference 16, and we also adopt this training strategy for our current work. We generated synthetic Poisson noisy images with the number of images, *N_x_*, at different noise levels, *x*%, following a Gaussian distribution centered on the mean noise value of 60%:

(2) $$N_{x}=N_{T}\int_{x-\delta /2}^{x+\delta /2}\frac{1}{\sqrt{2\pi }\sigma }e^{-\frac{{\left(x-60\right)}^{2}}{2\sigma^{2}}}dx$$

where *N*_T_ is the total number of training images, *δ* is the interval width, and *σ* is the standard deviation of the distribution, which was set to 20%.

### 2.2 Network architecture

**FIGURE 2:**
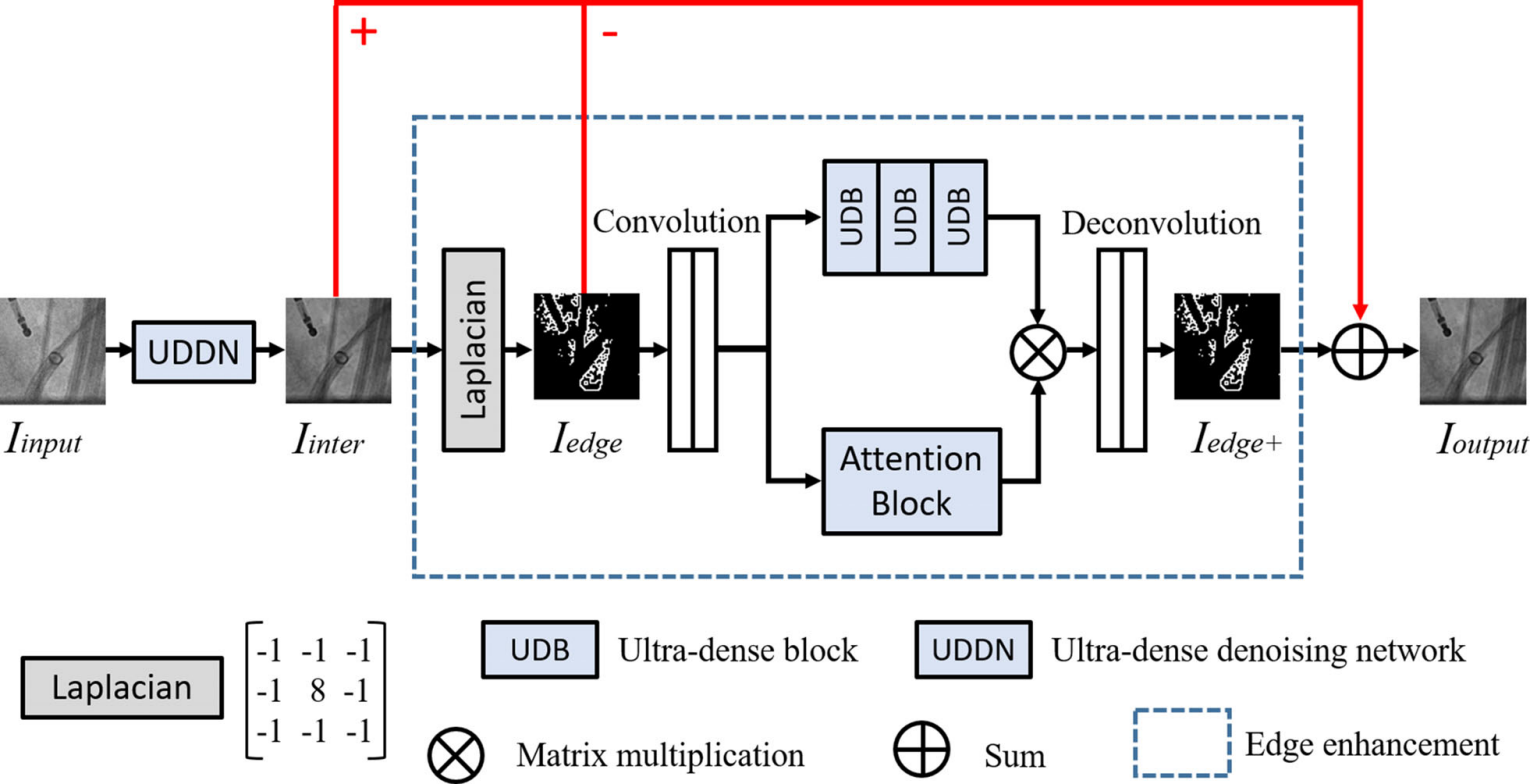
Outline of the proposed EEDN

As illustrated in Figure 2, the proposed framework consists of an initial denoiser and an edge‐enhancement subnetwork. First, an initial denoiser is used to generate an intermediate denoised result *I*_inter_. This block is similar to our previous UDDN but simplified in complexity to keep the overall network complexity the same. Second, we extract and enhance image contours of *I*_inter_ by compensating for fine edge information with an attention block and a group of interacted UDBs for edge feature representation. Finally, the intermediate result and enhanced edges can be combined to generate a new edge‐enhanced denoised image.

For the initial denoiser, we take the UDDN architecture design in reference 16 as a reference. Since the feature extraction performance of UDDN will reach a ceiling with the increase of UDBs, our initial denoiser has half the number of UDBs to reduce the computational burden. UDDN enables our framework to generate a denoised but marginally edge‐smoothed intermediate result as our edge extraction base. For edge enhancement, the Laplacian operator^22^ is used to extract the edge map *I*_edge_ of our intermediate result *I*_inter_. Then this edge map *I*_edge_ is enhanced to produce *I*_edge+_ through an attention‐awareness edge‐enhancement module. Finally, we generate a new denoised result *I*_output_ based on the previous result *I*_inter_ as follows:

(3) $$\;I_{\mathrm{inter}}+\left(I_{\mathrm{edge}+}-I_{\mathrm{edge}}\right)=I_{\mathrm{output}}$$

### 2.3 Edge enhancement

Edge enhancement aims to extract and enhance edge features instead of paying equal attention to all features. We design a specific attention mechanism based on the obtained edge map and propose an attention‐awareness edge‐enhancement module. The Laplacian operator is first utilized to label the image edges in *I*_inter_ before enhancement. The Laplacian *L* (*x*, *y*) of *I*_inter_ (*x*, *y*) can be defined from its second derivatives and is formulated as follows:

(4) $$L\left(x,y\right)=\frac{\partial^{2}\;I_{\mathrm{inter}}\left(x,y\right)}{\partial^{2}x}+\frac{\partial^{2}\;I_{\mathrm{inter}}\;\left(x,y\right)}{\partial^{2}y}$$

**FIGURE 3:**
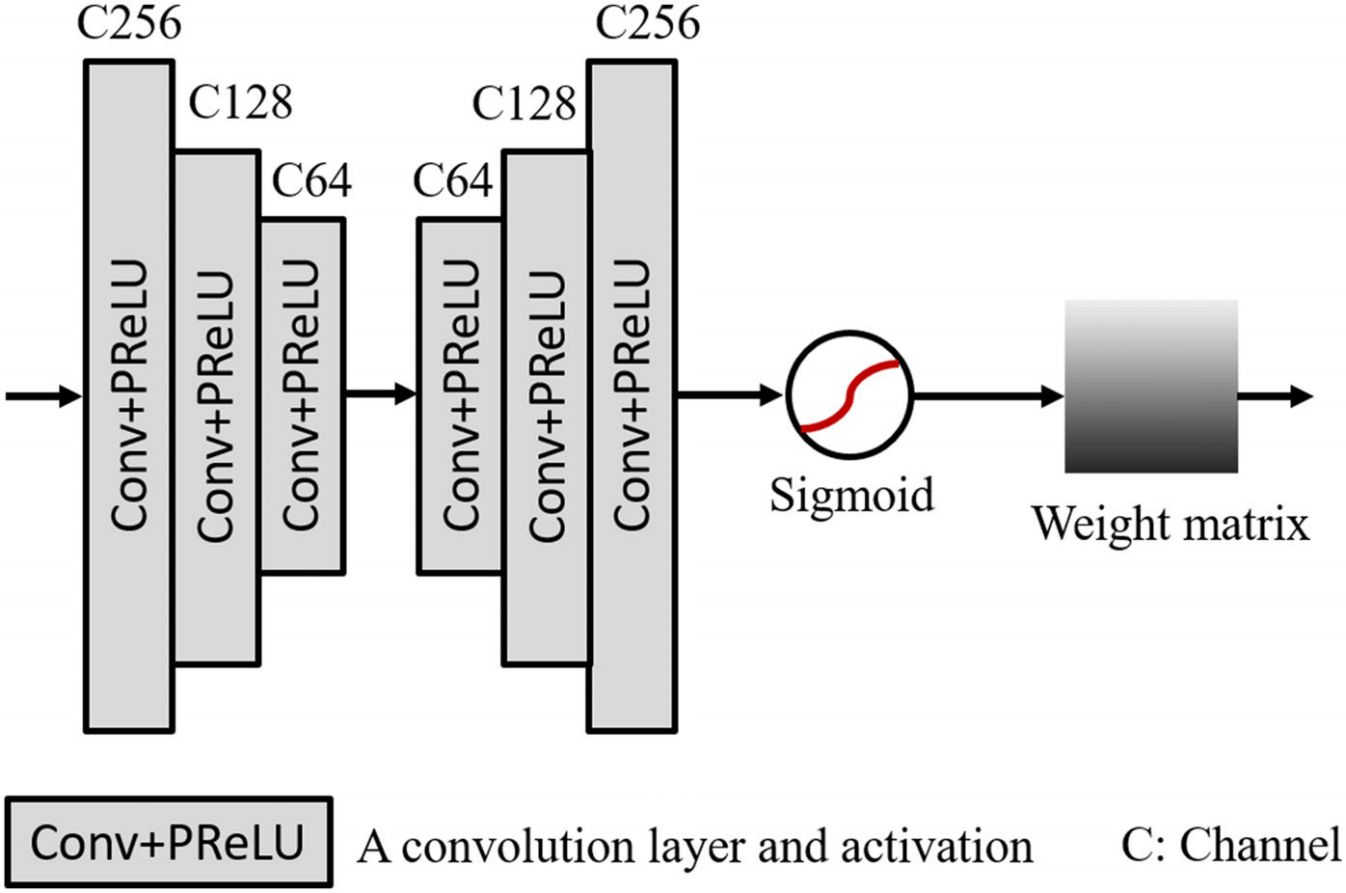
Outline of the attention block based on edge map

The Laplacian operator possesses isotropy and rotational invariance and produces a steep zero‐crossing point at edges. Accordingly, the edges can be determined and the edge map *I*_edge_ can be generated by convolving *I*_inter_ (*x*, *y*) with the Laplacian given in Equation (2). Then two stride convolution layers are added to map the extracted edge map to a low‐dimensional domain for reducing the calculation burden. Symmetrically, two deconvolution layers are added to map the edge features, jointly generated by multiple UDBs and an attention block, back to a high‐dimensional domain to obtain *I*_edge+_. On the one hand, UDBs designed in reference 16 are concatenated to extract the fine information based on the edge map. On the other hand, we simultaneously construct an attention block, as shown in Figure 3, to learn features with discrimination so that our module can be guided to focus on the real edge information. Our attention block has six stacked convolution layers (filter size is 3 × 3) followed by the activations, and this design enables a further feature extraction on edge information. After that, there is a sigmoid function worked as a threshold to provide learning discrimination. The weight matrix generated is used to highlight the edges and can be merged with the extracted results of the UDBs to generate a new map. At the end of our framework, we replace the over‐smooth edges in *I*_inter_ with the enhanced edge maps to obtain a more realistic denoising result.

### 2.4 Model optimization

According to previous CNN‐based image denoising methods,^9^, ^10^, ^11^, ^12^, ^13^, ^14^, ^20^ model optimization depends on iteratively minimizing the distance between the output image and the ground truth based on the feature level. As illustrated in Figure 1, *I*_output_ is our final denoising result and *I*’ represents the corresponding ground truth. In this framework, we use a loss function as follow:

(5) $$L_{\mathrm{content}}=\arg\min\sum_{i=1}^{n}\rho \left(I^{\prime }\left(i\right)-I_{\mathrm{output}}\left(i\right)\right)$$

where $\rho \left(x\right)=\sqrt{\left(x^{2}+\varepsilon^{2}\right)}$ represents the Charbonnier penalty function^23^ (a differentiable variant of the *l*_1_‐norm) and the compensation parameter *ε* is empirically set to 10^−3^ according to reference 16.

### 2.5 Evaluation

For image denoising, the evaluation of model performance on real‐world data is a difficult task, as image quality measurement is greatly affected by subjective opinions. Besides visual perception, extensive qualitative and quantitative evaluations should be performed to demonstrate the validity of a proposed method.

#### 2.5.1 Quantitative indicators

Similar to many previous representative denoising works,^9^, ^10^, ^11^, ^12^, ^13^, ^14^, ^16^, ^20^ we select two commonly used evaluation metrics, PSNR and SSIM, to validate the effectiveness of EEDN. Both need ground truth images for comparison, and the calculation of PSNR is based on mean squared error (MSE). For an *n*‐bit image *I* and its ground truth *I*’, its MSE and PSNR (in dB) can be calculated as

(6) $$\mathrm{MSE}=\frac{1}{M\times N}{\left|\left|I-I^{\prime }\right|\right|}^{2}$$

(7) $$\mathrm{PSNR}=10log_{10}\frac{{\left(2^{n}-1\right)}^{2}}{\mathrm{MSE}}$$

where *M* and *N* represent the width and height of *I* (*x*, *y*), respectively. SSIM can be calculated as

(8) $$\mathrm{SSIM}\left(I,I^{\prime }\right)=\frac{\left(2\mu_{I}\mu_{I^{\prime }}+c_{1}\right)\left(2\sigma_{II^{\prime }}+c_{2}\right)}{\left({\mu_{I}}^{2}+{\mu_{I^{\prime }}}^{2}+c_{1}\right)\left({\sigma_{I}}^{2}+{\sigma_{I^{\prime }}}^{2}+c_{2}\right)}$$

where *μ* is the mean pixel intensity, *σ* is the standard deviation/covariance, and *c_1 _* = *k_1_* (2*^n^*‐1) and *c_2 _* = *k_2_* (2*^n^*‐1), with *k_1 _* = 0.01 and *k_2 _* = 0.03 by default.

These evaluation metrics can only be used for the validation of model effectiveness based on synthetic X‐ray datasets which have ground truth, and we have only X‐ray fluoroscopy images with uncertain noise levels during interventions. In this paper, we chose local SNR for clinical dataset evaluation. The local SNR using image patches is calculated by taking the ratio of the mean pixel intensity, *μ*, to the standard deviation, *σ*, of the pixel intensity in each patch

(9) $$\mathrm{SNR}=\frac{\mu }{\sigma }$$

we then compute the mean local SNR by averaging all the patches in an image. The patch size was chosen to be 16 × 16 pixels.

#### 2.5.2 Frequency spectrum analysis

**FIGURE 4:**
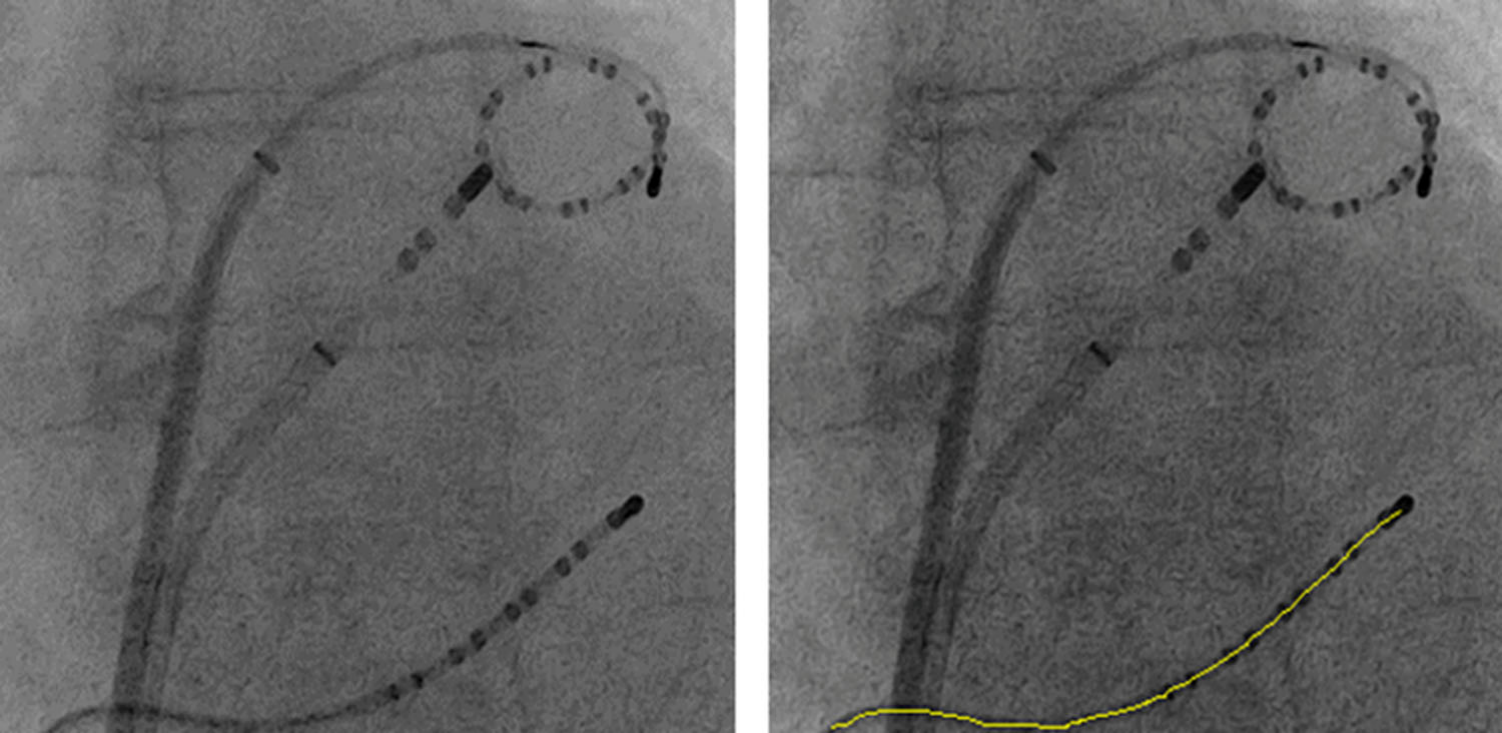
Example images from observer study to determine spatial frequencies of *useful content*. Left column: two examples from the CL2 dataset. Second column: log magnitude of Fourier transform of the original image. Third column: Results of bandpass filtering. Right column: User‐adjustable bandpass filter. The observer selected 0.051‐0.254 cycles/mm for the top image and 0.079‐0.289 cycles/mm for the lower image. The scales are the pixel coordinates from the top left corner

The frequency spectrum is commonly used to characterize the spatial frequency content of images. To further evaluate the edge restoration ability of our framework, we compared the results of EEDN to the results produced by the previous UDDN using frequency spectrum analysis. To compute the frequency spectrum of a single frame *I* (*x*, *y*), we first apply the Fourier transform to this image to obtain its representation in the frequency domain as:

(10) $$F\left(u,v\right)=\sum_{0}^{M-1}\sum_{0}^{N-1}I\left(x,y\right)e^{-j\left(\frac{ux}{M}+\frac{vy}{N}\right)}$$

where *M* and *N* represent the width and height of *I* (*x*, *y*), respectively. After this, we obtain the frequency power spectrum by

(11) $$S\left(r\right)=\frac{1}{n}\sum_{v}\sum_{u}\left|F\left(u,v\right)\right|\;\forall \;\;r\leq \sqrt{u^{2}+v^{2}}<r+1,\;$$

where *S* represents the average magnitude of spatial frequency r in *I* (*x*, *y*) and *n* is the number of elements in the annulus going from *r to r+1*. We then rescale the frequencies to cycles per millimeter using the Nyquist frequency determined from the X‐ray image DICOM header (using the detector element spacing, the source‐to‐image distance and the source‐to‐entrance distance). We compare the frequency spectra of X‐ray images generated by the different algorithms to understand their frequency transfer properties and examine their ability to preserve useful information. The useful information consists of anatomical cues, especially features of vertebrae, ribs, and the heart borders, and also the various devices that are being manipulated. In order to assess where in the frequency spectrum this information lies, we selected a sample of 10 clinical images (from the CL2 dataset, see Table 1) that contained a range of these features and asked five experienced observers to select lower and upper spatial frequency limits for each image that resulted in preservation of these features after bandpass filtering. A visual interactive interface was developed using Matlab that presented the original image, allowed the observers to select the lower and upper spatial frequencies of the bandpass filter and showed the image after bandpass filtering (Figure 4). The frequency limits were saved for all observers and processed to yield the overall range (minimum and maximum spatial frequencies of the entire dataset) and a range where all observers agreed, which we call the *consensus band*.

**TABLE 1:** Experimental dataset summary

**Dataset**	**Description**	**Devices/features of interest**	**Mean local SNR**	**Average Nyquist frequency (cycles/mm)**	**Network training**	**Network testing**
**CXR** ^a^	Standard diagnostic chest X‐ray	Standard features seen in a chest X‐ray	23.5	1.26	5000 images used to generate	300 images
**24**	30 805 patients 112 120 images 1024 × 1024 Frontal view	A mixture of no findings and pathologies	calculated from 100 random images		30 443 random patches (96×96) +Synthetic noise	300 central patches (576 × 576) +Synthetic noise
**CL1** ^b^	Left atrial radio‐frequency ablation 23 patients 23 fluoroscopy sequences 3.75‐7.5 fps 1013 images 512 × 512 PA, LAO30°	Coronary sinus catheter, standard radio‐frequency ablation catheter, lasso catheter, trans‐septal puncture needle	13.4 Calculated from 100 random images	1.0	800 images used to generate 10 554 random patches (96 × 96) + synthetic noise	Not used
**CL2** ^b^	Pacing study20 patients 100 fluoroscopy sequences 3.75 fps 3262 images 512 × 512 PA, RAO30°, LAO30°	Pacemaker box, standard pacing lead, temporary pacing wire, multi‐polar pacing wire, contrast injection	11.3 Calculated using the entire dataset	1.0	Not used	3262 images 3262 central patches (400×400)
**CD** ^b^	Left atrial radio‐frequency ablation 5 patients 8 fluoroscopy sequences 3.75‐7.5 fps 416 images 512 × 512 PA	Coronary sinus catheter, standard radio‐frequency ablation catheter, lasso catheter, trans‐septal puncture needle	12.2 calculated using the entire dataset	1.0	Not used	416 images 416 central patches (384∼432 × 384∼432)

*Abbreviations*:CD, catheter detection data; CL1, catheter laboratory data 1; CL2, catheter laboratory data 2; CXR, chestX‐Ray8.

^a^ The CXR dataset were acquired on various systems.

^b^ The CL1, CL2, and CD images were acquired on a Philips Allura Xper FD10 system.

#### 2.5.3 Clinician assessment

To evaluate and compare the denoising performance of the proposed EEDN with the previous UDDN, 10 X‐ray fluoroscopy sequences (consisting of 407 frames in total) from our clinical datasets (CL2, see Table 1) were used for clinical assessment by five cardiologists at St. Thomas’ hospital and the Brompton hospital, London. Three types of sequences were involved in the clinician assessment: the original X‐ray fluoroscopy sequences and the denoised results of UDDN and the new EEDN. This evaluation had to be performed remotely during the covid‐19 pandemic and was standardized as far as possible. Each sequence was formulated into a non‐compressed AVI file and pairs were presented side‐by‐side using a Microsoft Powerpoint presentation, each slide having the paired videos running synchronously. On the first slide there were a set of instructions. The cardiologists were told to view the images on 15‐inch screen at a distance of 1 m in a darkened room, that each pair of fluoroscopy sequences was generated using the same X‐ray dose, and that they should select the *preferred* sequence from the pair or select both if equally preferred. If one sequence was more acceptable a score of 1 was assigned to it and a score of 0 to the other. If both were given equal preference or no difference was observed, a score of 0.5 was assigned to each. There was a total of 40 paired comparisons. Ten pairs of sequences were identical to check for reliability in the cardiologists’ opinion. The remaining 30 pairs had cross‐comparison of each of the three different types of sequences, with each type appearing 20 times in total.

#### 2.5.4 Catheter detection evaluation

**FIGURE 5:**
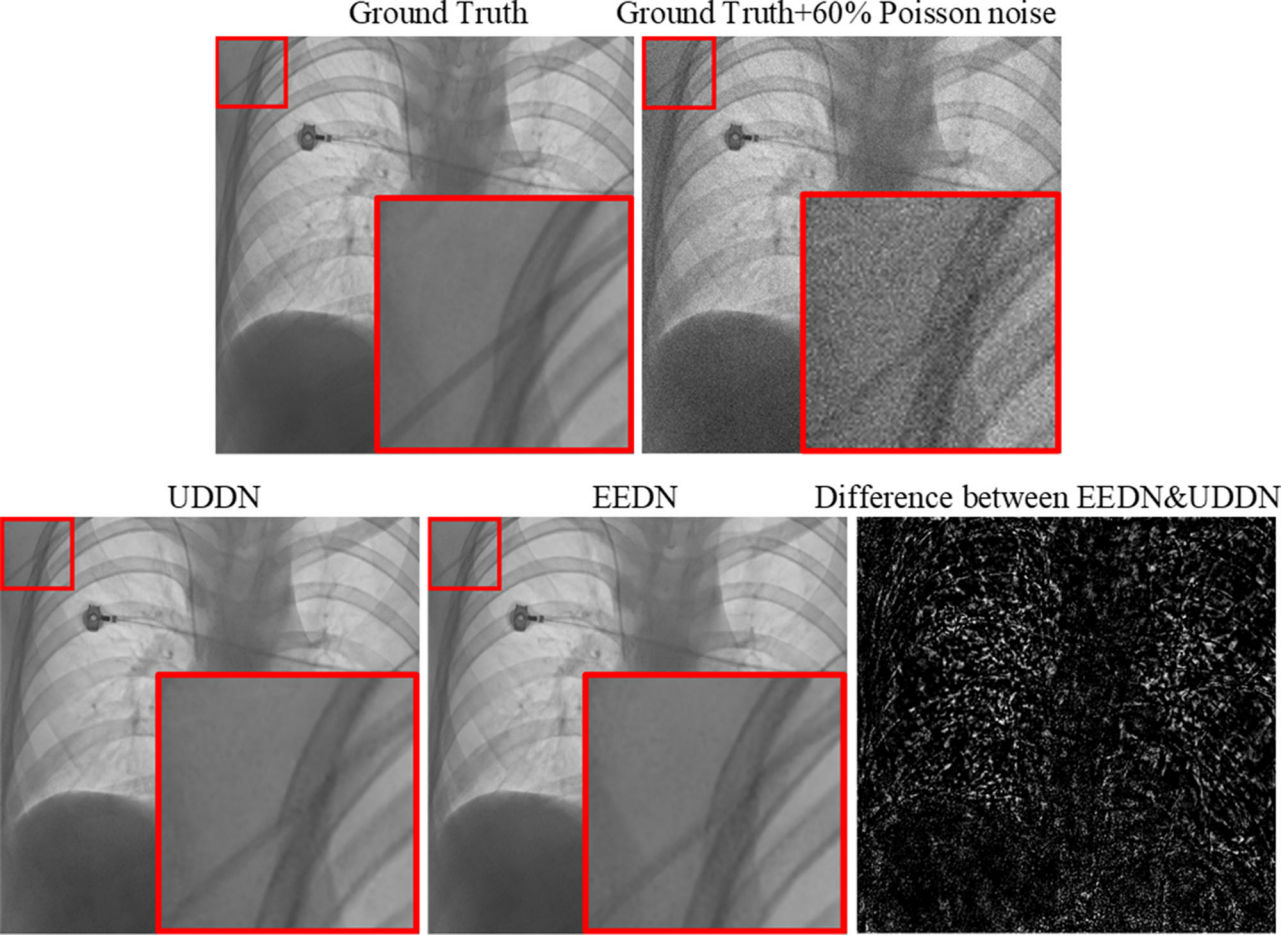
An example of catheter detection applied to a denoised X‐ray image by UDDN. The yellow line is the centerline of the detected CS catheter. Left: The denoised image. Right: The result of catheter detection

To validate that UDDN and the proposed EEDN do not deteriorate the performance of commonly applied computer vision algorithms, the catheter detection method in reference 24 was applied to 416 denoised X‐ray images (from the CD dataset, see Table 1) to extract the centerline of the CS catheter which was visible in each of these images. Figure 5 gives an example of catheter detection applied to denoised images. The detection error is defined as the average of shortest distances from points on the detected centerline to the corresponding annotated line, which was manually annotated by a clinical expert.^24^ The same catheter detection method then was applied to the original X‐ray images and the detection errors were also computed against the annotated lines.

## 3 EXPERIMENTS

In this section, we first introduce the basic experimental environment, including the experimental settings, X‐ray datasets, and model parameters (Section 3.1). After that, we compare EEDN with the previous UDDN using a synthesized Poisson noise dataset and PSNR and SSIM as quantitative indicators (Section 3.2). Then, our networks are tested on a clinical dataset using mean local SNR, frequency spectrum analysis, and clinician voting as quantitative indicators (Sections 3.3 and 3.4). Finally, we evaluate catheter detection applied on X‐ray images denoised by EEDN and UDDN as well as the original images (Section 3.5).

### 3.1 Datasets and setup

We performed our experiments using a publicly‐available dataset of chest X‐ray images, CXR,^21^ and three clinical X‐ray image datasets acquired at St. Thomas’ hospital during cardiac electrophysiology procedures. Table 1 shows the details of the data used for experiments. The clinical datasets were obtained during studies for which the patients gave informed consent for allowing the images to be used for research. The clinical images contained the usual anatomical structures seen in the thorax as well a variety of medical devices, such as pacing wires, electrophysiology catheters, pacemaker leads, pacemakers, sternal wires, ECG electrodes, and so on. The CL1 dataset consisted of 23 fluoroscopy sequences taken during left atrial ablation procedures. The CL2 dataset consisted of 100 fluoroscopy sequences taken during pacing studies. The CD dataset consisted of eight fluoroscopy sequences taken during left atrial ablation procedures, each having a CS catheter visible. Moreover, we calculated the mean SNR using Equation (9) to give an indication of relative dose for each of the datasets.

Based on the settings presented in reference 25, we inputted one batch consisting of 16 patches with the size of 96 × 96 from the training datasets (CXR & CL1) to our network each time. The CXR data was used for training because it has a high SNR and serves as a surrogate for *clean* images. The training was diversified by using a range of noise levels, as outlined in Section 2.1. and by including the clinical data from CL1. Although adding more and diverse training data is advantageous, it is limited by execution time. The learning rate was initialized to 10^−3^ for all layers and halved for every 2000 steps up to 10^−6^ and we selected PReLU^26^ as our activation following each convolution layer. To ensure a fair comparison, the number of UDBs in UDDN and EEDN are both 6 in total. In our experiments, we used a computer with an NVIDIA GTX1060Ti GPU with 6.0 Gb RAM, an Intel I7‐8700K CPU @ 3.20 GHz with 16.0 Gb RAM for training and testing. Our model was implemented on TensorFlow with Python3.6 under Windows10, CUDA9.0 and CUDNN5.1.

### 3.2 Validation

**FIGURE 6:**
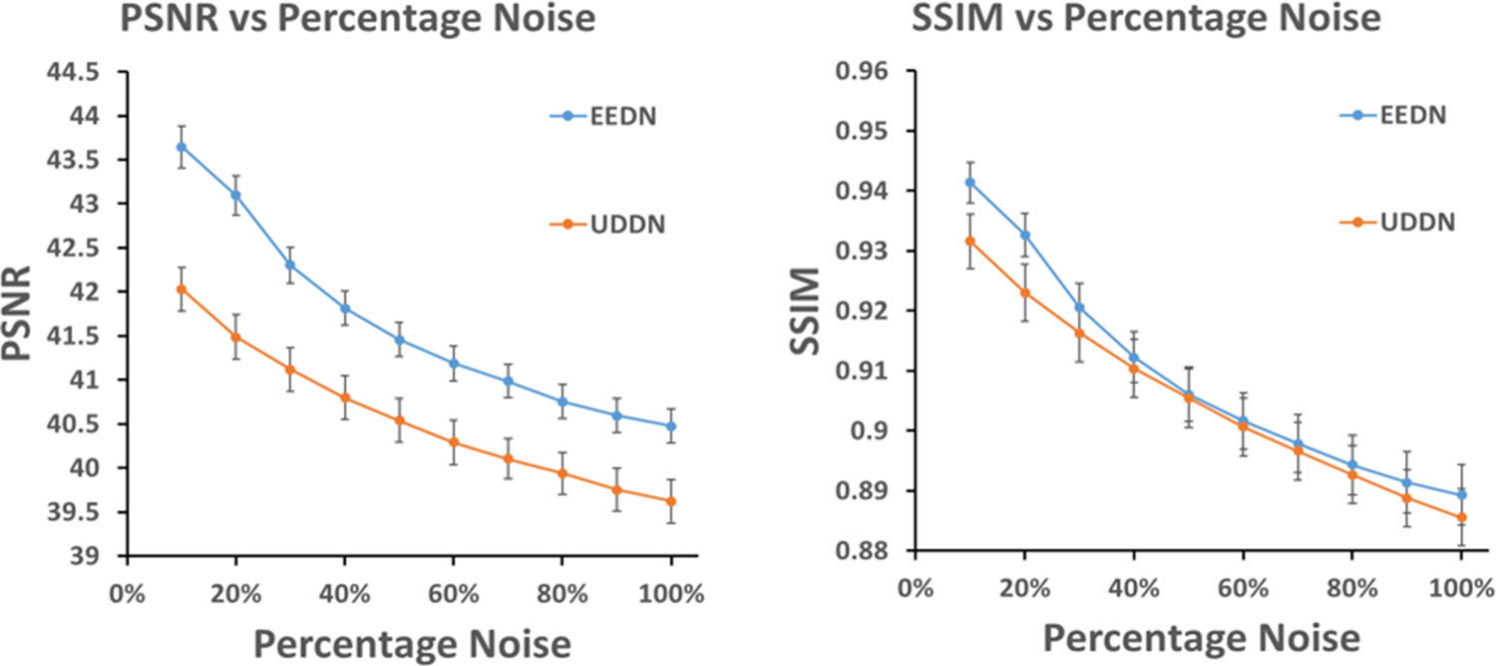
An example of the denoising results on the CXR test data using the noise level of 60%

In this part, we compared EEDN with UDDN,^16^ our previous network, which is a symmetrical architecture network stacked with six UDBs. The denoising results on two of the test chest X‐ray images created by adding fixed 60% noise are displayed in Figure 6. The ability of both networks to denoise is clearly evident but the differences are not easy to visually interpret. The difference image shows that networks do not differ in low spatial frequency regions, such as within the liver and the heart shadow, but do differ in edge regions. For the evaluation metrics (PSNR and SSIM) on the entire 60% added noise dataset, EEDN achieved a better PSNR and SSIM (41.50 dB and 0.9161), which were 0.15 dB and 0.002 higher than those of UDDN.

**FIGURE 7:**
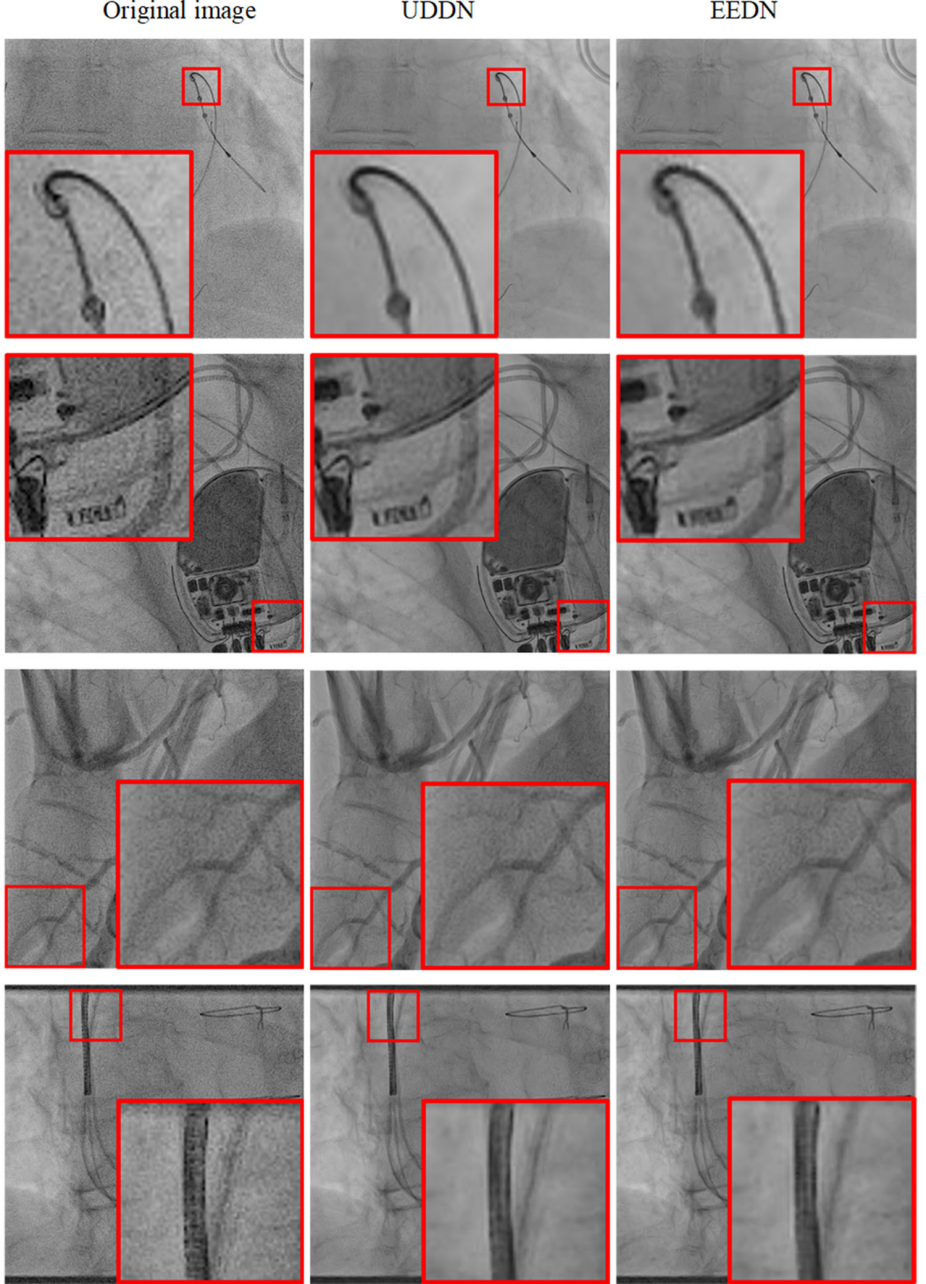
The PSNR and SSIM comparison of denoising results on the CXR test dataset at varying input noise levels using two CNN‐based methods: UDDN^16^ and the new EEDN. The number of sample images used to calculate each mean value was 30 and the error bars show the 95% confidence intervals

Furthermore, to validate the ability of the CNN‐based algorithms, we tested these denoising models with a range of noise levels from 10% to 100%. Figure 7 shows the results in terms of PSNR and SSIM. It is seen that EEDN exhibited higher PSNR and SSIM than UDDN. The differences in PSNR were significant over the entire noise level range but the differences in SSIM were only significant at the lower noise levels.

### 3.3 Clinical applications and analysis

**FIGURE 8:**
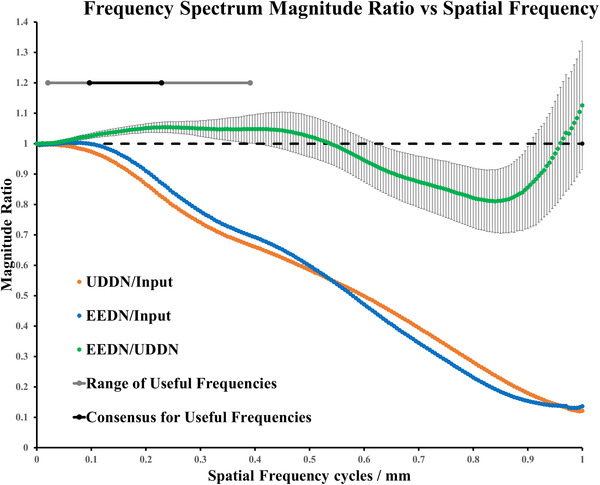
A visual comparison of denoising results on CL2 for UDDN and EEDN for four example images

The run time of EEDN is 0.17s/frame on average under the conditions of our equipment, which similar to that of UDDN. Four examples of denoised images from the CL2 dataset are shown in Figure 8. The ability of both networks to denoise the original images can clearly be seen. However, differences in denoising capability are difficult to interpret visually on single images and therefore these differences are better understood via the quantitative analysis and the clinical observations on dynamic denoised sequences. Table 2 shows the denoising results on the entire CL2 dataset in terms of the mean local SNR results and relative dose, and the relative dose is based on the assumption that SNR is proportional to the square root of dose. Both EEDN and UDDN showed statistically significant (*p* < 0.01) improvements in SNR when compared to the original images but there was no statistical difference between the EEND and UDDN results.

**TABLE 2:** Denoising results on the CL2 dataset

Algorithm	Mean SNR ± 1SD	Relative dose^a^
EEDN	24.5 ± 5.7	4.7
UDDN	24.6 ± 5.7	4.7
Original image	11.3 ± 1.5	1.0

*Abbreviation*:SNR, signal‐to‐noise ratio.

^a^ The relative dose is based on the assumption that SNR is proportional to the square root of dose.

**FIGURE 9:**
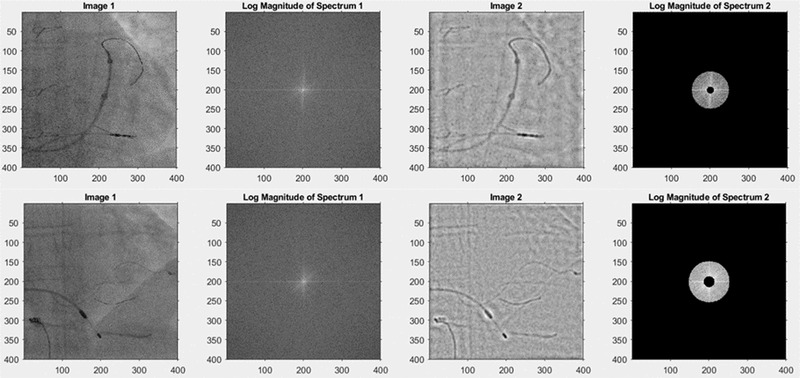
Mean frequency spectrum ratios. Error bars are shown for EEDN/UDDN using ±1SD. Note that the 95% confidence intervals for the mean values would be too small to be visible on these graphs since 3262 samples were used to calculate each mean ratio. The dotted black line is magnitude ratio = 1

The performance of these CNN‐based denoising methods cannot be evaluated comprehensively by using the mean local SNR metric alone. Therefore, we calculated the frequency spectrum for each image in the CL2 dataset, including input X‐ray fluoroscopy images, UDDN results and EEDN results. The Nyquist frequency for these images ranged from 0.93 to 1.22 cycles/mm. We computed the mean frequency spectrum by averaging over the entire 3262 images. We then computed the frequency magnitude ratios to characterize the frequency transfer of the UDDN and EEDN networks. From our observer study, we determined that useful information, in terms of anatomical cues and devices, lies between 0.02 and 0.39 cycles per mm (grey line on Figure 9). These were the extrema of the limits selected by our observers. All observers agreed that useful information lies between 0.10 and 0.23 cycles per mm (black line on Figure 9). Figure 9 shows the frequency transfer function of UDDN and EEDN when compared to the input data (orange and blue lines, respectively) and also the relative transfer function between UDDN and EEDN (green line). Both UDDN and EEDN cause increasing suppression of frequencies up to the Nyquist frequency. The green line shows that EEDN preserves frequencies in the useful band by providing a relative increase of up to 5% when compared to UDDN. This effect is likely due to the addition of the edge‐enhancement block in EEDN. There is relatively better suppression of frequencies above the useful band by EEDN when compared to UDDN. The trend in the error bars shows that there is more per‐image variability as spatial frequency increases. This would indicate that the frequency response of the networks is more consistent at lower spatial frequencies when compared to higher spatial frequencies. This would also indicate that the networks could not readily by modeled using a frequency transfer function and the response is image content dependent.

### 3.4 Clinician assessment

The voting scores were totaled across the five cardiologists at St. Thomas’ and the Brompton hospitals, and are presented in Tables 3 and 4. The reliability in Table 3 was assessed by using the percentage of correctly identified pairs of sequences that were identical. All cardiologists were deemed to have provided reliable voting, with the minimum reliability score being 80%. EEDN and UDDN were statistically preferred over the input sequences (*p* < 0.001). EEDN sequences were statistically preferred over UDDN sequences but with less significance (*p* = 0.07).

**TABLE 3:** Reliability and voting results on CL2 dataset per cardiologist

	Reliability (%)	Voting results
		UDDN: 14.5
Cardiologist 1	80	EEDN: 15.5
		Original image: 0
		UDDN: 11
Cardiologist 2	90	EEDN: 14
		Original image: 5
Cardiologist 3		UDDN:11
	90	EEDN: 14.5
		Original image: 4.5
Cardiologist 4		UDDN: 13
	90	EEDN: 15
		Original image: 2
Cardiologist 5		UDDN: 14
	80	EEDN: 15.5
		Original image: 0.5
Average		UDDN: 12.7
	86	EEDN: 14.9
		Original image: 2.4

**TABLE 4:** Voting results per sequence

	Original sequence	UDDN	EEDN
Original Image	0.60±0.50	UDDN > Input *p* < 0.001	EEDN > Input *p* < 0.001
UDDN		3.18 ± 1.29	EEDN > UDDN *p* = 0.07
EEDN			3.73 ± .99

*Notes*: The leading diagonal in shows the mean ±1SD voting score per sequence pair for each of original sequence, UDDN, and EEDN (*n* = 20), and the off‐diagonal elements show the results of hypothesis testing for difference in mean values using *t*‐tests.

### 3.5 Catheter detection

We further tested our models on a clinical catheter detection dataset (CD). The results are shown in Table 5. There was no statistical difference in any of the metrics, that is, mean error, failure rate, and detected length, between the original images and the denoised images from UDDN and EEDN. This indicates that application of these networks does not aid the task of catheter detection but also that it does not adversely affect this task.

**TABLE 5:** Catheter detection results on CD dataset

	Error ± 1SD (mm)			Failure rate (%)			Detected length (%)		
Sequence	Original	UDDN	EEDN	Original	UDDN	EEDN	Original	UDDN	EEDN
1	0.40 ± 0.07	0.36 ± 0.11	0.35 ± 0.10	0	0	0	92	94	95
2	0.51 ± 0.16	0.49 ± 0.24	0.52 ± 0.29	0	0	0	89	91	90
3	0.59 ± 0.23	0.55 ± 0.32	0.56 ± 0.31	0	0	0	95	96	96
4	0.73 ± 0.28	0.78 ± 0.34	0.77 ± 0.30	11	7	9	86	81	83
5	0.62 ± 0.17	0.64 ± 0.17	0.64 ± 0.23	7	7	7	84	88	82
6	0.74 ± 0.27	0.73 ± 0.25	0.76 ± 0.33	8	4	8	82	84	81
7	0.50 ± 0.09	0.50 ± 0.17	0.49 ± 0.14	0	0	0	94	95	94
8	0.53 ± 0.10	0.47 ± 0.17	0.43 ± 0.07	0	0	0	90	93	95
**Mean**	**0.58 ± 0.22**	**0.57 ± 0.28**	**0.57 ± 0.28**	**3**	**2**	**3**	**89**	**90**	**90**

## 4 CONCLUSION AND DISCUSSION

Recently, some attempts for X‐ray fluoroscopy sequence denoising have shown the potential for deep learning methods. In our previous work, we proposed a CNN‐based image denoising method, UDDN, which has achieved superior performance on catheter laboratory X‐ray data in terms of image SNR and clinician assessment when compared to other methods, for example, DnCNN. However, there were limitations to this method. According to the cardiologists interviewed, the denoised images by UDDN are sometimes too smooth, especially the edge information, which makes the X‐ray fluoroscopy sequences looks slightly artificial. To obtain X‐ray fluoroscopy sequences with less loss of useful information, we proposed a novel denoising framework, EEDN, in which an attention‐awareness edge‐enhancement module was designed to increase the edge sharpness. This framework was designed to extract and enhance the contours of X‐ray fluoroscopy images by optimizing their edge maps through an attention mechanism. Compared to our previous framework, EEDN provides an edge boost to an initial UDDN denoising result without increasing the total computational cost. To validate the effectiveness of EEDN, extensive qualitative and quantitative evaluations have been performed. For the synthesized Poisson noise dataset (CXR), EEDN achieved higher PSNR and SSIM than previous CNN‐based denoising method, UDDN, but the differences in SSIM were only significant at the lower noise levels. For clinical data (CL2), EEDN achieved a comparative SNR to UDDN, but it showed greater ability for preservation of useful information, as indicated by the frequency spectrum analysis. According to the perception of cardiologists, sequences denoised by EEDN are preferred than those denoised by the previous UDDN. For the catheter detection dataset (CD), EEDN does not significantly alter the results of this type of image processing.

Our current results are limited to application in cardiac electrophysiology procedures and application to other procedures where the X‐ray images may be from different X‐ray systems and with different dose settings, remains to be assessed. We hypothesize that this framework could be applied in other settings, especially since the network training can be diversified and adapted. We also aim to test the approach in real time in the catheter laboratory by implementing the moderate increase in execution speed that would be required to meet the typical frame rates that are used during electrophysiology procedures. Overall, this architecture provides a potentially useful approach to dose reduction for X‐ray guided cardiac interventional procedures.

## CONFLICT OF INTEREST

The authors declare no conflict of interest.
